# Metastatic carcinoma to the thyroid gland from renal cell carcinoma: role of ultrasonography in preoperative diagnosis

**DOI:** 10.1186/s13044-015-0016-4

**Published:** 2015-03-17

**Authors:** Kaoru Kobayashi, Mitsuyoshi Hirokawa, Tomonori Yabuta, Mitsuhiro Fukushima, Hiroo Masuoka, Takuya Higashiyama, Minoru Kihara, Yasuhiro Ito, Akihiro Miya, Nobuyuki Amino, Akira Miyauchi

**Affiliations:** Kuma Hospital, 8-2-35 Shimoyamate-dori, , Chuo-ku Kobe-City, 650-0011 Japan

**Keywords:** Thyroid, Ultrasonography, Metastatic carcinoma, Renal cell carcinoma, Diagnosis, Tumor thrombus

## Abstract

**Background:**

Patients with metastases to the thyroid from renal cell carcinoma (RCC) that need surgical management are not many and unfamiliar to clinicians and thyroid endocrinologists. Therefore, little information is available on ultrasonographic features of metastatic carcinoma in the thyroid. The strategic value of ultrasound in preoperative surgical planning for patients with thyroid nodules has become increasingly appreciated. The purposes of this article are to clarify the ultrasound characteristics of metastatic carcinoma to the thyroid from RCC by evaluating many patients in one institute, and to investigate the role of ultrasonography in preoperative diagnosis.

**Methods:**

Ten patients with these carcinomas who had undergone surgical management were investigated clinically and ultrasonographically. Ultrasonographic features to be evaluated were the form of involvement in the thyroid, size, shape, pattern, calcifications, vascularity, and tumor thrombus. Clinical features were previous history of RCC, serum thyroglobulin levels, cytology, preoperative diagnosis, and surgery.

**Results:**

Ultrasonographic features of these carcinomas were more likely to involve a solitary, irregular, and solid without calcifications, and prominent intra-tumoral vascularity and tumor thrombus in the vein. These patients tended to be older, and to have relatively late recurrence in the thyroid, RCC in the right kidney as the primary site, and relatively low serum thyroglobulin levels.

**Conclusions:**

Metastatic carcinomas to the thyroid from RCC presented highly characteristic features on ultrasonography. These ultrasonographic features combined with cytological findings and previous medical history of RCC can provide the optimal process for the preoperative diagnosis of such patients.

## Background

Metastases of renal cell carcinoma to multiple sites occur in the course of the disease and are generally regarded as the result of spread by a hematogenous route [1]. The thyroid gland is one of the metastatic sites from the primary lesion [1-3], but metastases to the thyroid that need surgical management are infrequent. It is reported that a surgical approach for such patients is associating with a favorable prognosis, when metastatic tumors are confined within the thyroid [1,3-6]. Therefore, preoperative diagnosis has clinical significance. Needless to say, previous medical history of renal cell carcinoma and fine-needle aspiration cytology of thyroid tumors of such patients are important for preoperative diagnosis.

The strategic value of ultrasound in preoperative surgical planning of patients with thyroid nodules has become increasingly appreciated [7,8]. The ultrasonogaphic features of a metastatic tumor to the thyroid from renal cell carcinoma are unfamiliar to clinicians and thyroid endocrinologists because of the rarity of such patients. It is unclear whether the ultrasonogaphic features can be helpful in the preoperative diagnosis, in addition to characteristic findings of cytology and previous medical history of renal cell carcinoma.

In this article, we focus on the ultrasonographic features of metastatic carcinomas to the thyroid from renal cell carcinoma, and discuss the significance of ultrasound for the preoperative diagnosis of such patients.

## Methods

We retrospectively reviewed the medical database of patients with thyroid malignancies who underwent surgery between January 1998 and December 2013 in Kuma Hospital. A total of 10 patients with metastatic carcinoma to the thyroid from renal cell carcinoma undergoing thyroid surgery were included in this study. Preoperative ultrasonography was performed in all patients who had thyroid surgery during this time period. The ultrasound readings that were used in this study were made as part of the care of the patients. Surgical samples of the thyroid, tumor thrombi, and lymph nodes were cut before fixation. Specimens were fixed in buffered formalin and embedded in paraffin, and HE and immunohistochemical staining was performed. All related pathological specimens were reviewed (by M.H.), and the histopathological diagnosis was included in this study. The preoperative ultrasound readings were confirmed visually by surgeons at surgery and histopathological examination postoperatively. The surgical findings and histopathological diagnosis were recorded in the medical database of the hospital. The ethics committee of Kuma Hspital approved the study protocol (US-RCC meta), which was in adherence to the Declaration of Helsinki.

Ultrasonographic examination was performed by well-trained, registered ultrasonographers, using a TOSHIBA Aplio SSA-770A ultrasound system with PLT-1204AX (7–14 MHz) and PLT-805AT (5–12 MHz) linear probes. We used both grayscale and power Doppler ultrasonography. Power Doppler ultrasonography was used predominantly to assess the vascularity of the thyroid tumor and to identify the presence or absence of tumor thrombus in the vein.

Age at thyroid surgery, sex, previous history of renal cell carcinoma, serum thyroglobulin level, anti-thyroglobulin autoantibody, and ultrasonographic findings of the thyroid tumor, fine-needle aspiration cytology, preoperative diagnosis, and surgery were investigated.

The ultrasonographic features of metastatic carcinomas to the thyroid from renal cell carcinoma to be estimated were as follows:

1. Form of involvement in the thyroid
  Tumor formation or diffuse involvement in the thyroid gland was estimated.
2. Location in the thyroid gland and size of tumors
  In terms of the right or left lobe, or the isthmus of the thyroid gland, the site where the tumor was located was estimated. The size of the tumor was recorded in mm.
3. Shape of tumor, tumor pattern, and strong echoes (calcifications)
  The shape of the tumor was classified into regular or irregular. The tumor pattern was classified into solid, mixed, or cystic. The presence or absence of strong echoes with acoustic shadowing (calcifications) was estimated within the tumor lesion.
4. Vascularity and distribution of blood signals
  The intensity of blood signals in the tumor was estimated by Doppler ultrasonography, and classified into -, ±, +, or ++. The distribution of blood signals was classified into intra-tumoral or peripheral dominant.
5. Extrathyroidal spread (swollen lymph node and tumor thrombus)
  The presence or absence of swollen lymph nodes in the neck and tumor thrombus in the thyroid veins and the internal jugular vein adjacent to the thyroid tumor was estimated.

## Results

### Clinical findings

There were 10 surgical patients with metastatic carcinoma to the thyroid from renal cell carcinoma in this period (Table 1). The histopathological diagnosis of the thyroid tumors was the clear cell variant of renal cell carcinoma in all of the 10 patients. One patient (Patient 7) revealed metastatic carcinoma in benign adenomatous nodule. The patients were 4 men and 6 women, and the age at thyroid surgery was 67.8 ± 7.3 years old (range, 57–79 years old; median, 68.5 years old). Eight patients presented with a previous medical history of nephrectomy for renal cell carcinoma 13.4 ± 8.4 years earlier (range, 2–30 years; median, 12.5 years). In two patients (Patients 9 and 10), renal cell carcinoma in the right kidney was discovered as a primary carcinoma after the thyroid surgery and the histopathological diagnosis of metastatic carcinoma to the thyroid gland. The side of renal cell carcinoma was the right in nine patients (Patients 1–6, and 8–10) and the left in one patient (Patient 7). One patient (Patient 5) revealed positivity for anti-thyroglobulin auto-antibody. Serum thyroglobulin (normal range, < 40 ng/ml) was 136. 9 ± 285.2 ng/ml (range, 9.3-894.6 ng/ml; median, 30.2 ng/ml) in the other 9 patients without auto-antibody.

**Table 1:** **Patients with metastatic carcinoma to the thyroid from renal cell carcinoma and ultrasonographic features**

**Patient**		**History, Side of RCC**	**Serum Tg, TgAb**	**Ultrasonographic features of metastatic carcinoma**				**FNAC**	**Pre-op. DX**	**Surgery**
**No**	**Age, Sex**			**Location, Size (mm)**	**Shape, Pattern, Calc.**	**Intra-tumoral vascularity**	**Tumor thrombus**			
1	70F	8 yr, Right	81.3 (-)	Rt, 36x19x36	irregular solid, (-)	++	(-)	malignant, meta/RCC	meta/RCC	LO
2	77M	9 yr, Right	9.3 (-)	Rt, 56x35x48	irregular solid, (-)	++	(-)	indeterminate	meta/RCC	LO
3	72M	30 yr, Right	27.4 (-)	Lt, 31x19x24	irregular solid, (-)	++	(-)	benign, AN	meta/RCC	LO
4	58F	10 yr, Right	68.2 (-)	Rt, 39x23x36	irregular solid, (-)	++	(+)	indeteminate	meta/RCC	LO+RTV+RIJV
							TV-IJV			
5	67F	18 yr, Right	128.5 (+)	Rt, 81x41x70	irregular solid, (-)	++	(+)	malignant, meta/RCC	meta/RCC	TT+RTV+RIJV
				Lt, 27x16x18		++	TV-IJV			
6	63F	2 yr, Right	894.6 (-)	Rt, 48x29x30 +LNS	irregular solid, (-)	++	(-)	malignant, meta/RCC	meta/RCC	LO+MND
7*	57M	15 yr, Left	26.1 (-)	Rt, 48x40x43	irregular solid, (-)	+	(+)	malignant, meta/RCC	meta/RCC	LO+RTV
							TV			
8	79F	15 yr, Right	24.5 (-)	Rt, 45x33x34	irregular solid, (-)	++	(-)	benign, AN	AN	LO
9	65F	(-)** Right	70.7 (-)	Rt, 23x20x21	regular solid, (-)	+	(-)	fol. tumor, FT	FT	LO
10	70M	(-)** Right	30.2 (-)	Rt, 25x19x19	irregular solid, (-)	++	(+)	benign, AG	AG or PC?	TT+CND+RTV
				Lt, 12x5x10			TV			

A previous history of renal cell carcinoma was recognized in 7 patients (Patients 1-7) before surgery, and in 3 patients (Patients 8-10) it was not. Age: age at thyroid surgery, F: female, M: male, Rt: right, Lt: left, History: previous history of nephrectomy for renal cell carcinoma, RCC: renal cell carcinoma, Tg: thyroglobulin (normal range: < 40 ng/ml), TgAb: anti-thyroglobulin autoantibody. Shape of the tumor was classified into regular or irregular. Pattern of the tumor was classified into solid, mixed, or cystic. Calc.: intra-tumoral calcification; (+) or (-). Intra-tumoral vascularity was classified into -, +, or ++ according to the intensity of blood signals by Doppler ultrasonography. FNAC: fine-needle aspiration cytology, Pre-op. DX: preoperative diagnosis, meta/RCC: metastatic carcinoma to the thyroid from renal cell carcinoma, LO: lobectomy of the thyroid, TT: total thyroidectomy, MND: modified neck dissection, CND: central node dissection, TV: thyroid vein, IJV: internal jugular vein RIJV: partial resection of internal jugular vein, RTV: resection of thyroid vein, LNS: lymph node swelling in the neck, FT: follicular tumor, AN: adenomatous nodule, AG: adenomatous goiter, PC; papillary carcinoma. Patient 7*: Metastatic renal cell carcinoma in benign adenomatous nodule. The benign part of the tumor could not be detected on ultrasonography because of small size. (-)**: Existence of renal cell carcinoma was recognized after the thyroid surgery and the histopathological diagnosis of thyroid tumor.

### Ultrasonographic features

#### Form of involvement in the thyroid

**Figure 1:**
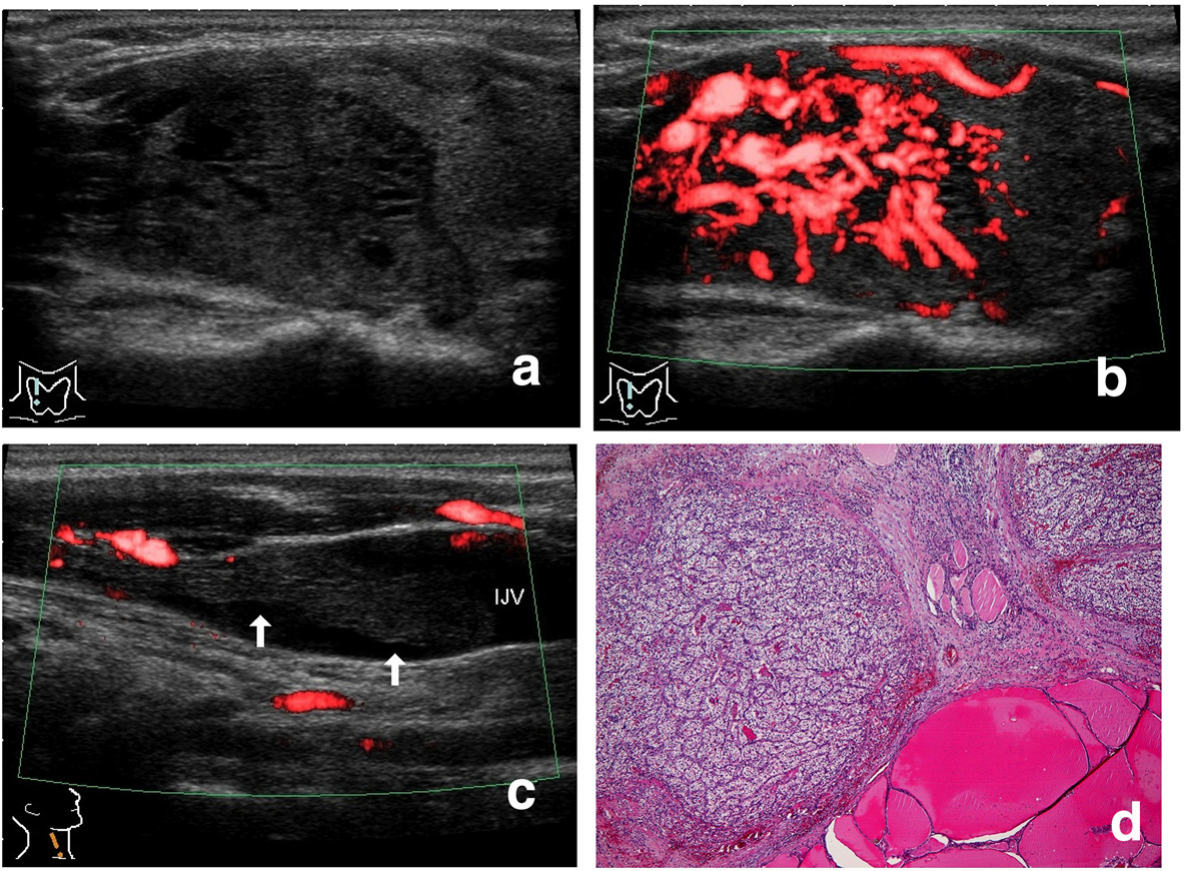
**(Patient 4). a**. Ultrasonography of the right lobe. A solid tumor. The margins are well demarcated and irregular, and the internal echo is predominantly hypoechoic and partially anechoic. Strong echoes with acoustic shadowing (calcifications) are not present. **b**. Power Doppler ultrasonography of the same section as in Figure 1a. Marked chaotic vascularity is predominantly shown in the intra-tumoral lesions. **c**. Power Doppler ultrasonography of the right internal jugular vein. A solid mass (arrows), that is, tumor thrombus, is shown as an “echogenic tongue” in the lumen. **d** Hematoxylin & eosin staining. Metastatic carcinoma of the clear cell variant of renal cell carcinoma and normal thyroid tissue are shown. Tissue of the tumor histopathogically demonstrates a pseudo-alveolar or pseudo-follicular structure and no calcifications.

**Figure 2:**
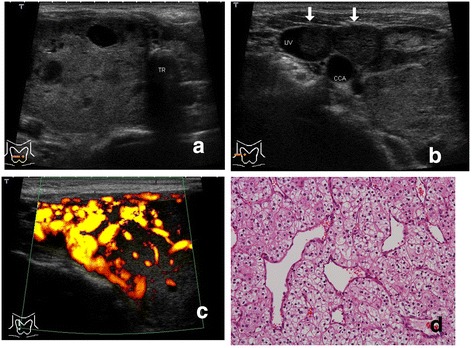
**(Patient 5). a**. Ultrasonography of the right lobe. A predominantly solid tumor. Internal echo shows homogenity in the solid area and interspersed cystic-like areas. (Power Doppler showed intense blood signals in these cystic-like areas). **b** Ultrasonography of the right lobe and the jugular vein. A solid mass (arrow on the left), that is, tumor thrombus, is shown in the lumen of the jugular vein. The lumen of the middle thyroid vein is completely occupied with tumor thrombus (arrow on the right). **c**. Power Doppler ultrasonography. Marked chaotic vascularity. **d** Hematoxylin & eosin staining. A rich vascular network.

**Figure 3:**
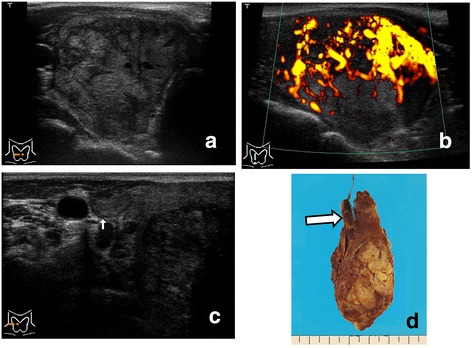
**(Patient 7). a**. Ultrasonography of the right lobe. A solid tumor. The internal echo is iso- to hypo-echoic and heterogeneous, and appears to show multiple small masses in the tumor. **b**. Power Doppler ultrasonography. **c**. Ultrasonography of the right lobe. Tumor thrombus (arrows) is observed in the superior thyroid vein. **d**. Gross resected specimen. Solid tumor in the right lobe of the thyroid and bulbous intravascular component, tumor thrombus (arrow), are shown.

Metastatic carcinomas showed tumor formation in the thyroid in all 10 of the patients (Figures 1, 2 and 3), and did not show diffuse involvement in the thyroid.

#### Location and size of thyroid tumor

The location of metastatic tumors was the right lobe of the thyroid gland in 9 patients, and the left lobe in 3 patients (Patients 3, 5, and 10). Two patients (Patients 5 and10) presented two tumors in the right and left lobes of the thyroid gland. The maximum size of the tumors was 39.3 ± 18.2 mm (range, 12–81 mm; median, 37.5 mm).

#### Shape of tumor, tumor pattern, and calcifications

The shapes of the tumors were irregular and well demarcated, and the tumor pattern showed a solid and hypoechoic level without a capsule-like structure and direct contact to the normal thyroid tissue in all 10 of the patients. None of the tumors demonstrated strong echoes with acoustic shadowing (calcifications) within them.

#### Vascularity and distribution of blood signals

The intensity of vascularity in the tumors by Doppler ultrasonography was ++ in 8 patients (Figures 1b, 2c, 3b) and + in 2 patients. Blood signals were more dominant in intra-tumoral lesions than in peripheral lesions of the tumors in all of the patients.

#### Extrathyroidal spread (swollen lymph node and tumor thrombus)

One patient (Patient 6) showed swollen lymph nodes in the neck in addition to the thyroid tumor. Direct tumor extension into the adjacent veins, that is, tumor thrombi, was detected in 4 patients preoperatively. Tumor thrombi within the internal jugular vein via the thyroid vein (“echogenic tongue”) were demonstrated in two patients (Patients 4 and 5) (Figures 1c, 2b), and those within the thyroid veins in two patients (Patienta 7 and 10) (Figure 3c). Intravascular components of the lesions appeared as a solid and hypoechoic mass without calcifications (Figures 1c, 2b, 3c).

### Fine-needle aspiration cytology (FNAC)

FNAC showed metastatic carcinoma to the thyroid from renal cell carcinoma in 4 patients, indeterminate in 2 patients (Patients 2 and 4), benign in 3 patients (Patients 3, 8, and 10), and follicular neoplasm in one patient (Patient 9).

### Preoperative diagnosis

Among the eight patients who had a previous history of nephrectomy for renal cell carcinoma, seven patients were accurately diagnosed with metastatic carcinoma to the thyroid from renal carcinoma preoperatively, and one patient (Patient 8) was diagnosed with a benign thyroid tumor. Two patients (Patients 9 and 10) who did not have a previous history of nephrectomy for renal cell carcinoma were misdiagnosed with follicular tumor and benign tumor, or papillary carcinoma preoperatively as the primary tumors.

### Surgery

Total thyroidectomy was performed in 2 patients and lobectomy in 8 patients. Resection of thyroid veins or partial resection of internal jugular veins was performed in 4 patients because of tumor thrombi in the veins (Patients 4, 5, 7, and 10). Central and modified neck lymph node dissections in addition to thyroid lobectomy were performed in two patients (Patients 6 and 10).

## Discussion

Table 1 reveals that these patients tend to be older, and to have relatively late recurrence in the thyroid, renal cell carcinoma in the “right” kidney as a primary site, and relatively low serum thyroglobulin levels. Three patients (Patients 8–10) could not be correctly diagnosed preoperatively, although 7 patients (Patients 1–7) were identified to have metastatic carcinoma. The previous history of renal cell carcinoma could not be obtained preoperatively in one patient (Patient 8). In 2 patients (Patients 9 and 10), thyroid metastasis was the initial presentation of the renal cell carcinoma. It is of interest that ultrasonography played an important role in preoperative diagnosis.

Thyroid ultrasound is particularly useful for estimating the location of a nodule, its size, and its solid or cystic nature, and also for guiding the performance of fine-needle aspiration biopsies. Therefore, it has already become established as the diagnostic procedure of choice in guidelines [7,8] for the management of thyroid nodules by every professional organization of endocrinologists. Papillary thyroid carcinoma is the most common and primary malignancy, and ultrasound features of such patients, for example, a predominantly solid, hypoechoic, ill-circumscribed nodule and lymph node swelling in the neck [9], have already become familiar. On the other hand, patients with metastases to the thyroid from renal cell carcinoma that need surgical management are not many and unfamiliar to clinicians and endocrinologists. It is reported that a surgical procedure for thyroid metastasis from renal cell carcinoma has benefits for the prognosis of such patients [1,3-6]. Little information is available on the ultrasonographic features of metastatic tumor to the thyroid from renal cell carcinoma [2,10-13]. For the reasons mentioned above, ultrasound features of metastatic carcinoma to the thyroid from renal cell carcinoma should be clarified by the evaluation of many patients. A full understanding of ultrasound features is clinically important and essential for such patients’ care.

The most common primary site from which metastasis to the thyroid gland occurs is reported to be the kidney in a clinical series [1,2]. Renal cell carcinoma arises from the renal tubular epithelial cells, and the clear cell type is the most common variant [14]. Histopathologically, clear cell carcinoma shows polygonal cells with clear cytoplasm, distinct cell membranes, and small compact eccentric nuclei, and a rich vascular network [14]. The propensity of primary renal cell carcinoma in the kidney to spread to regional lymph nodes and virtually every organ site is well recognized [14]. Furthermore, this carcinoma often invades regional veins and forms tumor thrombus in the lumen of the renal veins or the inferior vena cava. In addition, this carcinoma behaves unpredictably and its recurrence after nephrectomy is highly variable, presenting as late metastases several years after the initial surgery [1-3,5,10,12,13]. In this situation, metastatic carcinomas in the thyroid occur as a result of hematogenous spread from renal cell carcinoma [14].

A pseudo-alveolar or pseudo-follicular pattern of clear cell carcinoma may resemble the clear cell variant of thyroid carcinoma and adenoma histopathologically. Recently, it has been described that metastatic tumor in the thyroid should be diagnosed based on rich vascularity, abundant extravasated erythrocytes, pseudofollicular spaces filled with blood, negativity for thyroglobulin, and positivity for CD10 and positivity for the renal cell carcinoma marker carbonic anhydrase IX [15]. Histopathological features of the thyroid tumors inevitably reflect ultrasound images of these tumors. Sonographers and endocrinologists must be aware that the ultrasound images of thyroid tumors should be read and understood on the basis of the histopathological features of the tumors.

According to our series (Table 1), metastatic carcinomas to the thyroid from renal cell carcinoma formed one or two nodules within any portion of the thyroid glands in all 10 of the patients. No patients revealed diffuse involvement in the thyroid such as diffuse sclerosing variant of papillary thyroid carcinoma or diffuse swelling of Hashimoto’s thyroiditis on histopathology. Furthermore, these tumors did not form a distinct capsule of the tumor histopathologically, and neoplastic cells of these tumors were directly exposed to the normal thyroid tissue and showed invasiveness to adjacent tissue (Figure 1d). For that reason, these tumors showed an irregular margin without a capsule-like structure and directly contacted the normal thyroid region on ultrasonography (Figures 1, 2 and 3).

The tissue of these tumors histopathologically demonstrated a pseudo-alveolar or pseudo-follicular structure (Figures 1d, 2d), and cystic degeneration did not occur within the tumor. Additionally, calcifications did not form within these tumors, and there was a very rich vascular network in the tumor parenchyma (Figure 2d). In terms of the echogenicity of the internal echo of these tumors, they were hypoechoic compared with normal thyroid tissue, which reflected the homogenity of the tissue of these tumors on histopathology. These tumors did not demonstrate cystic anechoic lesions with posterior acoustic enhancement, or any type of echogenic foci with acoustic shadowing, that is, calcifications, on ultrasonography. Doppler ultrasonography showed intensive blood signals or prominent chaotic vascularity in the intra-tumoral area, but did not show prominent vascularity in the peri-tumoral area (Figures 1b, 2c, 3b).

Neoplastic cells of these tumors directly invaded intra-tumoral vessels and formed tumor thrombi in the lumen of the vessels. Grayscale and Doppler ultrasonography showed an “echogenic tongue”, that is, tumor thrombus, in the lumen of the thyroid vein or the internal jugular vein (Figures 1c, 2b, 3c) in 4 of the 10 patients. We previously reported on tumor thrombus in surgical patients with thyroid malignancies [16]. Of the 5507 patients with thyroid malignancies, there were 9 patients with tumor thrombi. (Two of these previously reported patients are Patients 4 and 5 in Table 1 of this article.) It can be said that metastases to the thyroid from renal cell carcinoma have a high frequency of tumor thrombus as extrathyroidal spread on ultrasonography. In the literature, tumor thrombi were also reported in a small number of patients with metastatic carcinoma from renal cell carcinoma [4,10,17]. Tumor thrombi in veins adjacent to the thyroid gland can be described as a noteworthy ultrasonographic feature because they are highly suggestive of malignancy. We believe that the detection of tumor thrombus by ultrasonography is useful for preoperative determination of the surgical procedure for such patients [4,10,17] as well as for the diagnosis of metastasis from renal cell carcinoma.

In one patient (Patient 7), small nest of benign adenomatous nodule were demonstrated in the peripheral tissue of the tumor histopathologically, although the majority of the tumor was occupied with clear cell carcinoma. This tumor revealed metastatic renal cell carcinoma in benign adenomatous nodule. The benign part of the tumor could not be detected on ultrasonography preoperatively because of small size. It is reported that renal cell carcinoma rarely metastasized to primary thyroid tumors [18].

There are some ultrasonographic classification systems for thyroid nodules. Among them, Kuma Hospital’s ultrasound classification system (Kuma’s USC, USC 1–5) [19,20] and the Thyroid imaging reporting and date system (TIRADS, TIRADS 1–6) [21] are popular and useful for clinical management at present. These ultrasound classification systems represent the risk of malignancy of thyroid nodules. When these two ultrasound classification systems are applied to the tumors in these 10 patients, they are classified in USC 4 (probably malignant) and 5 (malignant) on Kuma’s USC, and TIRADS 5 (probably malignant) and 6 (malignant) on the TIRADS classification. In particular, tumors with tumor thrombi (Patients 4, 5, 7, and 10) can be easily classified into USC 5 and TIRADS 6.

Quasi-static and shear-wave elastography now become available for diagnostic ultrasonic procedures in addition to conventional grayscale and Doppler ultrasonography. Quasi-static elastography is a relatively new technology that maps the relatively elastic properties of soft tissues, and shear-wave elastography is a new technology that shows quantitative stiffness value of tissues by measuring the velocity of wave propagation. Currently, Adamczewski et al. reported the findings of these two different elastography techniques in a patient with metastases of renal clear-cell carcinoma to the thyroid [22]. Both techniques revealed differences in relative stiffness (strain ratio) between the normal tissue and metastases. However, the absolute stiffness values for metastases in shear-wave elastography were within the range characteristic benign lesions. The data accumulation of these new techniques will add to the interpretation of conventional ultrasound images, and be helpful for ultrasonic diagnosis.

Thyroid metastasis from renal cell carcinoma has some characteristic ultrasonographic findings, such as prominent chaotic intra-tumoral vascularity and tumor thrombus as mentioned above; however, these findings are not specific to this disease. It is thus necessary to perform ultrasonography and fine-needle aspiration biopsy and to obtain information on the previous history of renal cell carcinoma in order to make a correct diagnosis preoperatively. It is reported that fine-needle aspiration cytology can suggest the possibility of metastatic tumor from renal cell carcinoma, and is essential for preoperative diagnosis [1,3,23]. Cytological findings of thyroid metastasis from renal cell carcinoma tend to be bloody, a lack of colloid, and striped nuclei with only occasional cells showing clear cytoplasm [23]. When clear cells are identified within the thyroid gland, primary tumors with clear cell features including papillary, follicular, and medullary thyroid carcinoma, and secondary tumor such as those from lung and salivary gland also need to be considered.

When a previous history of nephrectomy for renal cell carcinoma is recognized in such a patient, ultrasound examination as well as cytology is effective and helpful in order to diagnose it preoperatively. Actually, three patients (Patients 2, 3, and 4) showed an “indeterminate” or “benign” status because of too many blood cells and no neoplastic cells in cytology. However, these patients had a previous history of nephrectomy for renal cell carcinoma and demonstrated characteristic ultrasonographic findings for metastatic carcinoma from renal cell carcinoma. Therefore, we could make the correct preoperative diagnosis. In contrast, when we do not recognize a previous history of renal cell carcinoma, or thyroid metastasis is the first symptom as renal cell carcinoma in a patient, it seems to be difficult to diagnose it preoperatively because of the rarity of the disease. Unfortunately, two patients (Patients 9 and 10) who had no previous history of renal cell carcinoma were not correctly diagnosed preoperatively in spite of the existence of characteristic features on ultrasonography. Preoperative recognition of a previous history of renal cell carcinoma in a patient must be the most important clue for the correct diagnosis of the disease, which many articles in the literature have emphasized [1,2,6].

Cytology was falsely negative in four patients with previous history of renal cell carcinoma (Patients 2, 3, 4, and 8). We should have measured thyroglobulin in the washout of fine-needle aspiration cytology. When thyroglobulin was low or detectable, clinical suspicion of metastatic nodule would become more definitely.

## Conclusions

Comprehensive judgment of ultrasonographic findings of thyroid tumor, cytological findings, and previous medical history of renal cell carcinoma are suggested to lead to the correct diagnosis of patients with metastases to the thyroid from renal cell carcinoma. In this situation, ultrasound is the initial examination and such characteristic features on ultrasonography mentioned above can be clues to the correct diagnosis.

### Consent

Written informed consent was obtained from the patient’s guardian/parent/next of kin for the publication of this report and any accompanying images.

## References

[1] Chung AY, Tran TB, Brumund KT, Weisman RA, Bouvet M (2012). Metastases to the thyroid: a review of the literature from the last decade. Thyroid.

[2] Sindoni A, Rizzo M, Tuccari G, Ieni A, Barresi V, Calbo L (2010). Thyroid metastases from renal cell carcinoma: review of the literature. Scientific World Journal.

[3] Hegerova L, Griebeler ML, Reynolds JP, Henry MR, Gharib H: Metastasis to the thyroid gland: Report of a large series from the Mayo Clinic. *Am J Clin Oncol* doi: [10.1097/COC.0b013e31829d1d09] 10.1097/COC.0b013e31829d1d09 23799287

[4] De Stefano, Carluccioo R, Zanni E, Marchiori D, Cicchetti G, Bertaccini A (2009). Management of thyroid nodules as secondary involvement of renal cell carcinoma: case report and literature review. Anticancer Res.

[5] Assouad J, Banu E, Brian E, Pham DN, Dujon A, Foucault C (2008). Strategies and outcomes in pulmonary and extrapulmonary metastases from renal cell cancer. Eur J Cardiothorac Surg.

[6] Duggal NM, Horattas MC (2008). Metastatic renal cell carcinoma to the thyroid gland. Endocr Pract.

[7] Gharib H, Papini E, Paschke R, Duick DS, Valcavi R, Hegedues L (2006). AACE/AME/ETA Task Force on Thyroid Nodules: American Association of Clinical Endocrinologists and Associazione Medici Endocrinologi for clinical practice for the diagnosis an management of thyroid nodules. Endocrine Pract.

[8] Cooper DS, Doherty GM, Haugen BR, Kloos RT, Lee SL, American Thyroid Taskforce on Thyroid Nodules and Differentiated Thyroid Cancer (2009). Revised American Thyroid Association management guideline for patients with and differentiated thyroid cancer. Thyroid.

[9] Ahuja AT: Head and Neck. Differentiated thyroid carcinoma; In *Diagnostic Imaging: Ultrasound*, ed 1, Salt Lake City, Utah. AMIRSYS: 2007; 6–11.

[10] Pickhardt PJ, Pickard RH (2003). Sonography of delayed thyroid metastasis from renal cell carcinoma with jugular vein extension. Am J Roentgenol.

[11] Kim AY, Park SB, Choi HS, Hwang JC (2007). Isolated thyroid metastasis from renal cell carcinoma. J Ultrasound Med.

[12] Kihara M, Yokomise H, Yamauchi (2004). Metastasis of renal cell carcinoma to the thyroid gland 19 years after nephrectomy: a case report. Auris Nasus Larynx.

[13] Wada N, Hirakawa S, Rino Y, Hasuo K, Kawachi K, Nakatani Y (2005). Solitary metachronous metastasis to the thyroid from renal clear cell carcinoma 19 years after nephrectomy: report of a case. Surg Today.

[14] Russo P (2000). Renal cell carcinoma: presentation, staging, and surgical treatment. Semin Oncol.

[15] Cimino-Mathew A, Sharma R, Netto GJ (2011). Diagnostic use of PAX8, CAIX, TTF-1, and TGB in metastatic renal cell carcinoma of the thyroid. Am J Surg Pathol.

[16] Kobayashi K, Hirokawa M, Yabuta T, Fukushima M, Kihara M, Higashiyama T (2011). Tumor thrombus of thyroid malignancies in veins: Importance of detection by ultrasonography. Thyroid.

[17] Matei DV, Brescia A, Nordio A, Spinelli MG, Melegari S, Cozzi G (2001). Renal cell carcinoma and synchronous thyroid metastasis with neoplastic thrombosis of the internal jugular vein: report of a case. Arch Ital Urol Androl.

[18] Bohn OL, De las Casas LE, Leon ME (2009). Tumor-to-tumor metastasis: Renal cell carcinoma metastatic to papillary carcinoma of thyroid-report of a case and review of the literature. Head Neck Pathol.

[19] Yokozawa T, Miyauchi A, Kuma K, Sugawara M (1995). Accurate and simple method of diagnosing nodules the modified technique of ultrasound-guided fine needle aspiration biopsy. Thyroid.

[20] Ito Y, Amino N, Yokozawa T, Ota H, Ohshita M, Murata N (2007). Ultrasonographic evaluation of thyroid nodules in 900 patients: comparison among ultrasonographic, cytological, and histological findings. Thyroid.

[21] Horvath E, Majlis S, Rossi R, Franco C, Niedmann JP, Castro A (2009). An ultrasonogram reporting system for thyroid nodules stratifying cancer risk for clinical management. J Clin Endoccrinol Metab.

[22] Adamczewski Z, Dedecjus M, Skowronska-Jozwiak E, Lewinski A (2014). Metastases of renal clear-cell carcinoma to the thyroid: a comparison of shear-wave and quasi-static elastograhy. Pol Arch Med Wewn.

[23] Bhalla R, Popp A, Nassar A (2007). Case report: metastatic renal carcinoid to the thyroid diagnosed by fine needle aspiration biopsy. Diagn Cytopathol.

